# Anti-Cancer Peptides: Status and Future Prospects

**DOI:** 10.3390/molecules28031148

**Published:** 2023-01-23

**Authors:** Gehane Ghaly, Hatem Tallima, Eslam Dabbish, Norhan Badr ElDin, Mohamed K. Abd El-Rahman, Mahmoud A. A. Ibrahim, Tamer Shoeib

**Affiliations:** 1 Department of Chemistry, The American University in Cairo, New Cairo 11835, Egypt; 2 Analytical Chemistry Department, Faculty of Pharmacy, Cairo University, Kasr-El Aini Street, Cairo 11562, Egypt; 3 Department of Chemistry and Chemical Biology, Harvard University, 12 Oxford Street, Cambridge, MA 02138, USA; 4 Computational Chemistry Laboratory, Chemistry Department, Faculty of Science, Minia University, Minia 61519, Egypt; 5 School of Health Sciences, University of Kwa-Zulu-Natal, Westville, Durban 4000, South Africa

**Keywords:** anticancer peptides, cancer therapy, peptide conformation, peptide mode of action, cancer microenvironment

## Abstract

The dramatic rise in cancer incidence, alongside treatment deficiencies, has elevated cancer to the second-leading cause of death globally. The increasing morbidity and mortality of this disease can be traced back to a number of causes, including treatment-related side effects, drug resistance, inadequate curative treatment and tumor relapse. Recently, anti-cancer bioactive peptides (ACPs) have emerged as a potential therapeutic choice within the pharmaceutical arsenal due to their high penetration, specificity and fewer side effects. In this contribution, we present a general overview of the literature concerning the conformational structures, modes of action and membrane interaction mechanisms of ACPs, as well as provide recent examples of their successful employment as targeting ligands in cancer treatment. The use of ACPs as a diagnostic tool is summarized, and their advantages in these applications are highlighted. This review expounds on the main approaches for peptide synthesis along with their reconstruction and modification needed to enhance their therapeutic effect. Computational approaches that could predict therapeutic efficacy and suggest ACP candidates for experimental studies are discussed. Future research prospects in this rapidly expanding area are also offered.

## 1. Introduction

Various approaches to cancer treatment have been proposed and investigated; however, many limitations persist, including toxic side effects, developed drug resistance and low selectivity. These limitations motivated the search for new non-conventional methods in cancer therapy, of which anti-cancer bioactive peptides (ACPs) demonstrated potential in diagnostic and therapeutic applications, making them a good prospect for theranostic agents. In therapeutic applications, ACPs have demonstrated higher specificity, sensitivity, accuracy and lower toxicity than conventional anticancer therapeutics [1]. ACPs were also applied in combined therapy to increase cancer cell sensitivity to other therapeutic agents [2].

Peptides are small bioactive proteins, ranging between 10 and 100 amino acid units, that perform various biochemical roles in the body [3,4]. They are linked by peptide bonds that are formed by dehydration and condensation. The application of peptides in medicine was first proposed in 1922 through the use of insulin extracted from animal pancreas in the treatment of type 1 diabetes [5]. To date, more than 600 peptides have been applied in clinical and preclinical investigations, of which 60 have been approved as drugs [6,7]. These therapeutic applications of these peptides include cancer treatment, drug-delivery systems, hormonal regulators, inflammation modulators, vaccines, antibiotics and quorum-sensing molecules [8,9,10,11,12,13]. Peptides that have therapeutic effects are classified into three main categories based on their source: natural, artificially modified and artificially synthesized [14,15]. They can be obtained from living organisms, such as animals, plants, bacteria and fungi. They can also be attained through proteolysis, synthesized chemically or by recombinant genes [16,17]. Recently, computational methods such as traditional machine learning (ML) and deep learning (DL), which are a subset of artificial intelligence (AI), have been employed in ACPs screening [13,18,19] in order to overcome potential adverse in vitro effects due to peptidases or unknown immunogenicity [20].

Antimicrobial peptides (AMPs) are part of the innate immunity in numerous organisms [21]. They are typically cationic, amphipathic molecules with high content of hydrophobic residues. These properties allow them to interact, within a short time frame, with negatively charged microbial membranes that have a low probability of developing AMP resistance, resulting in microbial death [22,23]. A group of AMPs has also displayed anticancer activity, and hence they are also regarded as ACPs and are summarized in Table 1, which lists all ACPs discussed in this review. This anticancer activity is most likely due to their interactions with the higher abundance of negatively charged molecules, such as phosphatidylserine, glycoproteins and glycolipids, on the outer plasma membranes in cancer cells relative to their counterparts in normal cells [24].

## 2. Conformations of ACPs

### 2.1. ACPs with α-Helical Conformations

Most ACPs adopt α-helical conformations; for example, BMAP-27 and BMAP-28 both adopt this conformation and are both bovine cathelicidin-derived AMPs with demonstrated antitumor activity against leukemia [26,49]. They are composed of 27 and 28 amino acid residues, respectively, where the first 18 residues from their NH_2_-termini form amphipathic α-helices, while the remaining residues form hydrophobic tails, a crucial feature for their cytotoxic activity [50]. Another example is P18, where its α-helical COOH-terminus has been shown to be responsible for selective anticancer activity towards human cancer cells, including Jurkat T leukemia, K562 chronic myeloid leukemia and MDA-MB-361 breast carcinoma cells with no hemolytic effects [45].

The peptide leucine-37 (LL-37) belongs to the cathelicidin family and is encoded by the CAMP gene with a highly conserved NH_2_-terminal α-helix. It was initially synthesized as the preproprotein, hCAP-18, then converted into its active form, LL-37, by proteinase 3-mediated extracellular cleavage [51]. The preproprotein hCAP-18 is expressed in various cell types, including neutrophils [52] and squamous epithelial cells [53]. The active form, LL-37, plays a crucial role in adaptive immunity, growth inhibition, chemotaxis and wound healing [42,54,55,56].

Cecropin A and B are ACPs first discovered in insects such as the giant silk moth *Hyalophora cecropia* [57], then later in mammals [58]. The insect-derived peptides consist of 34-39 amino acid residues [30,59]. These peptides are each composed of two α-helices. Cecropin A has an NH_2_-terminal helix that is highly amphipathic, while the carboxylic terminal helix is hydrophobic. Cecropin B1, on the other hand, has two amphipathic helices and exhibits high antitumor activity against HL-60 human promyelocytic leukemia cells and low toxicity toward normal cells [60].

Some other α-helical ACPs, such as magainins and their analogs, gaegurins, aurein 1.2 and citropin 1.1, have been isolated from amphibian skin [25,31,34,61,62,63]. Magainins consisting of 21–27 amino acid residues, with separate cationic and hydrophobic faces within their helices, have been isolated from the African frog *Xenopus laevis* [64]. The most common ACP, magainin 2, and its synthetic analogs, magainins A, B and G, all show lytic activity against hematopoietic and solid tumor cell lines at concentrations 5–10 fold lower than that affecting normal cells [62,65]. These ACPs have also demonstrated antitumor activity against the human lung cancer cell line A59 [66] and against several human bladder cancer lines through the formation of ion-conducting pores in cell membranes [62,67].

Gaegurins are a class of six peptides exhibiting cytotoxic activities that have been isolated from the skin of the Korean frog *Rana rugose* [68]. These peptides acquire a random coil conformation in solution but revert to an α-helix in membrane environments. Gaegurin 5 and 6 each consist of 24 amino acid residues and possess selective anticancer cytotoxicity with minimal effect on normal cells [34,35,61]. Gaegurin 5 and two of its synthetic analogs have shown selective antitumor activity against HCT116 colon and MCF-7 breast carcinoma cells [34]. Similarly, gaegurin 6 and its synthetic analog, PTP7, have shown selective and broad antitumor activity against various human cancer cells and, more importantly, against a multidrug-resistant variant of the breast cancer MCF-7 cell line. This demonstrated action is thought to be via an apoptotic mechanism, as evident in the DNA fragments detected in the cell line environment after being treated with the peptides [61].

Aurein 1.2 and citropin 1.1 are isolated from the Australian frog *Litoria raniformis* and the tree frog *Litoria citropa,* respectively. Both are short peptides consisting of 13 and 16 amino acid residues, respectively. Aurein 1.2 acquires α-helical conformation in solution and has shown moderate anticancer activity against almost 60 human cancer cell lines, with no significant lytic effect on erythrocytes [25]. Citropin 1.1 has an α-helical structure with well-defined hydrophobic and hydrophilic regions. This peptide exhibits a wide range of antitumor activity against human hematopoietic and non-hematopoietic cancer cell lines, with no significant lytic effect on erythrocytes [31].

Melittin is a 26 amino acid residue peptide isolated from the venom of the European honeybee *Apis mellifera* [69]. Starting from the NH_2_-terminus, 19 out of 20 residues are hydrophobic, whereas the six residues ending at the COOH-terminus are hydrophilic for an overall alkaline peptide [70,71]. At low membrane concentrations, melittin adopts an α-helical structure parallel to the lipid bilayer [44]. The activity of melittin has been shown against both cancer cells and normal erythrocytes [70,71,72].

### 2.2. ACPs with β-Sheet Conformations

The second most common geometric arrangement for ACPs is β-sheet conformations [73,74]. Adopting this conformation are defensins, which are a group of Cys- and Arg-rich closely related ACPs, ranging from 29 to 45 amino acid residues [75], where some of its plant-derived are reported to have activity on cancer cells [76]. In this group of ACPs, three intramolecular disulfide bridges form between the NH_2_-terminal and COOH-terminal regions and are formed by six conserved Cys residues. In human α- and β-defensins, the disulfide bridges occur from the NH_2_-terminal between Cys1–Cys6, Cys2–Cys4 and Cys3–Cys5 in α-defensins, and between Cys1–Cys5, Cys2–Cys4 and Cys3–Cys6 in β-defensins [77,78,79]. This results in a cyclic, triple-stranded, amphiphilic β-sheet structure with separated hydrophobic and hydrophilic regions [80,81]. The human neutrophil peptides HNP 1, 2 and 3 are α-defensins, originally purified from the azurophilic granules of neutrophils [39,82]. These three ACPs exhibited their activity against several types of cancers, including the pro-monocytic human myeloid leukemia cell line U937, the human erythroleukemic cell line K562, and lymphoblastoid B cells IM-9 and WIL-2 [83].

Lactoferricin is another ACP adopting a β-sheet conformation that is obtained through the pepsin-mediated hydrolysis of mammalian milk, iron-binding and glycoprotein lactoferrin [84]. The bovine peptide LfcinB consists of 25 amino acid residues, with one disulfide bridge linking the two terminal regions of the peptide. It has two amphipathic structures, a loop peptide and a twisted β-sheet configuration that is attained in solution [85,86], with segregation of the basic amino acids on one face and the hydrophobic amino acids on the other [41]. LfcinB has demonstrated wide anticancer activity, including against human and mouse leukemia, fibrosarcoma and neuroblastoma cells, as well as other carcinomas [85,87,88,89], with no toxicity towards normal cells at the applied peptide concentrations [89,90]. LfcinB also suppresses both the basic fibroblast growth factor and the vascular endothelial growth factor-driven proliferation as well as migration of human endothelial cells [91].

Tachyplesin I, a 17 amino acid residue peptide isolated from the horseshoe crab *Tachypleus tridentatus* [48], is arranged in two parallel β-sheets joined together by two disulfide bridges. This geometry exposes six basic Arg and Lys amino acid units on the peptide surface to give it an amphipathic structure [92]. The Cys residues maintain the stability of the peptide in serum, with no effect on its cytotoxic action [93].

### 2.3. Linear, Hybrid, Diastereomeric and Synthetic ACPs

Brevinin, a 25 amino acid residue peptide identified in the skin secretions of the Fujian large-headed frog, *Limnonectes fujianensis*, was predicted to be an amphipathic, hydrophobic, alpha helical and beta turn peptide that has the ability to penetrate membrane lipid bilayers [28]. PR-39, on the other hand, a Pro- and Arg-rich linear peptide of the cathelicidin family containing 39 amino acid residues with no secondary structure, is isolated from the porcine small intestine and neutrophils [94,95]. Hybrid ACPs, on the other hand, are a class of synthetic peptides created by combining different regions of different peptides, for example, the positively charged NH_2_-terminal α-helical region of cecropin A with the NH_2_-terminal α-helical hydrophobic region of either melittin or magainin 2 [96,97,98]. Both hybrids have shown anticancer effects against lung cancer cell lines. Modest hemolytic activity was demonstrated by the melittin hybrid, while the magainin hybrid has exhibited little or no lytic effect on erythrocytes [96]. The central hinge region (Gly–Ile–Gly) was reported to be crucial for anticancer activity, as it provides the required conformational flexibility allowing for interactions between the α-helical NH_2_-terminus and cell membranes. This flexibility leads to parallel alignment of the peptides, which in turn permits the insertion of the α-helical COOH-terminus through cell membranes [98].

Synthetic peptides are able to permeabilize cancerous cell membranes without being degraded enzymatically in serum [99,100]. D-K4R2L9 is a synthetic, 15 residue, diastereomeric amphipathic peptide, with D-amino acids making up a third of its sequence while also containing Leu, Lys and Arg residues. This synthetic peptide has shown anticancer activity against mouse melanoma cell lines, human prostate cancer cell lines, and significant prevention ability against lung tumor formation [101]. D-K6L9, another 15-residue synthetic diastereomeric amphipathic peptide, with D-Lys and D-Leu residues in one-third of its sequence, showed selective anticancer activity against human prostate cancer cell lines similar to D-K4R2L9 [33]. L-K6L9, an analog of D-K6L9, which consists of L-amino acids only, also showed similar anticancer activity; however, it also caused lysis of normal fibroblast and erythrocytes [102]. Magainins A, B and G which are synthetic analogs of magainin, all show significant antitumor cytotoxic activity against lung cancer and drug-resistant tumor cells [62,66,103,104]. It is noteworthy that the hybrid peptide formed by linking cecropin with magainin 2 has shown remarkable anticancer activity against several cancer cell lines, with low toxicity to erythrocytes and fibroblasts [105]. The synthetic derivatives of LfcinB, with clear cationic and hydrophobic sectors, show higher anticancer activity than their natural analog. The glutamic acid-containing murine peptide, on the other hand, has shown no anticancer activity, suggesting that the high net positive charge plays a major role in the demonstrated anticancer cytotoxicity [106,107,108]. Synthetic ACPs are typically coupled to targeting domains to further promote their selectivity and cytotoxic activity. For example, cyclic CNGRC and double cyclic RGD-4C have both been coupled to a mitochondrial membrane targeting 14 D-amino acid residues (a repeating KLAKLAK unit), pro-apoptotic peptide [109].

## 3. Modes of Action of ACPs

Some ACPs are active against microbial and cancerous cells, such as cecropins and magainins, while other ACPs are additionally active against normal cells, such as melittin and human neutrophil defensins HNP-1, HNP-2 and HNP-3 [72,73,110]. In most cases, the main action of ACPs is through their interactions with cell membranes, resulting either in their lysis or in their penetration. However, in a small number of cases, some other minor mechanisms also occur [24]. Interactions of ACPs with membranes involve several factors that promote tumor cell membrane charge modification and electrostatic interactions between typically cationic peptides and highly negatively charged cell membranes. Hypoxia and elevated levels of reactive oxygen species modify tumor microenvironments, disrupting phospholipid symmetrical distribution between the inner and outer plasma membrane layers. This exposes the highly expressed anionic phosphatidylserine on the outer layer, allowing for recognition by cationic ACPs [111,112]. High concentrations of phosphatidylethanolamine zwitterions, deregulation of glycosylation, glycolipids and membrane glycoproteins with repeated regions of O-glycosylation, in addition to over-expression of heparan sulfate proteoglycans, all contribute to the acquired negative charge of a tumor cell membrane [111,113]. Other factors such as an increased number of filopodia and microvilli, which provides more surface area for contact [114]; lower cholesterol content and rigidity of the tumor plasma membrane, exposing it to hydrophobic interactions [115,116], also contribute to selective ACPs cytotoxicity towards tumor cell membranes.

Several models describing the interactions of ACPs with cell membranes have been investigated. Those models have been elucidated through various techniques, including circular dichroism, X-ray crystallography, nuclear magnetic resonance, reverse-phase high-performance liquid chromatography and surface plasmon resonance [117,118].

### 3.1. Membrane Interaction Mechanisms

#### 3.1.1. The Carpet Model

**Figure 1:**
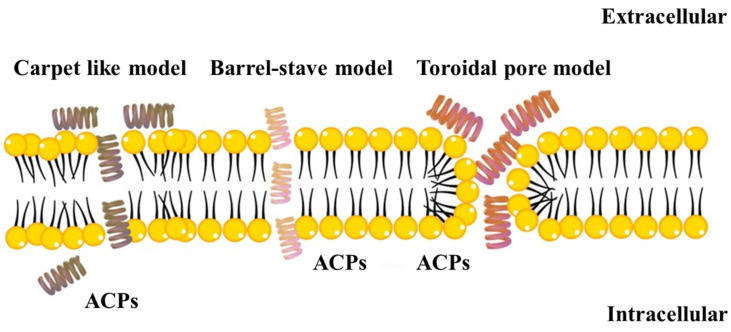
Schematic representation of the most significant models of ACPs action. The yellow circles and the black linkers represent the hydrophilic and hydrophobic regions, respectively, of the phospholipid cell membranes.

In the so-called carpet model, the α-helical ACPs accumulate on the plasma membranes of cells through electrostatic interactions in a parallel fashion similar to a carpet, as shown in Figure 1. After reaching a critical concentration, the ACPs rotate on themselves, redirecting the membrane phospholipids. Similar to a detergent effect, this results in increased membrane fluidity, destruction of the lipid bilayer and micelle formation. The cells’ plasma membranes rupture due to the strain exerted on them, causing their eventual death due to their penetration by the ACPs [73,119]. Defensins are an example of ACPs acting through this model [120]. Magainins and their analogs, gaegurins, aurein 1.2 and citripin 1.1, also adopt this mechanism through this model at low concentrations [120], with the latter two ACPs adopting other mechanisms when they dimerize [121,122].

#### 3.1.2. The Barrel-Stave Model

The initial accumulation of ACPs in their monomeric forms on cell plasma membranes, then as their local concentration increases, as barrel-shaped multimers, is the namesake hallmark of this model. This induces conformational changes in the lipid bilayer, exposing the hydrophobic core to the hydrophobic amino acids in the peptides, which weakens the cell membranes and forms transmembrane pores through the hydrophilic peptide sections, as shown in Figure 1. This, in turn, leads cells to leake their contents and cause their subsequent death [116,123]. The ACPs LfcinB, the helical alamethicin, the dimers of each of aurein, citropin 1.1 and melittin peptides, through this mode of action, form transmembrane pores that disturb membrane integrity, allowing for their entrance into the cells cytoplasmic compartments, and for their co-localization with the negatively charged mitochondria causing cell death [85,124]. Gaegurins were also reported to achieve their cytolytic effect is achieved through the barrel stave and or the carpet model [125].

#### 3.1.3. The Toroidal Pore Model

The toroidal pore model is a multi-step model, where initially, the ACPs are disposed of parallel to the lipid bilayers, reverting to their active form upon reaching a threshold concentration. This allows the ACPs to attain a perpendicular position over the bilayers, destabilizing the membraned of the cells and forming within them toroidal pores that enable the peptides to reach their inner membrane leaflets. This, in turn, is followed by pore disintegration, allowing the peptides into the cells compartments, as presented in Figure 1 [119,126]. Inside the cells, ACPs inhibit essential pathways, such as DNA replication or protein synthesis, causing cells termination. Cecropin A, magainin 2, protegrin-1 and LL-37 are examples of ACPs acting through the toroidal pore mechanisms [116,127]. LL-37 has also been shown to be selective in mitochondrial depolarization and cause caspase-independent apoptosis in human oral squamous cell carcinoma SAS-H1 cells [128,129].

#### 3.1.4. Other Minor Models

There are several other minor mechanisms that are in common with the action of AMPs and allow for ACPs to interact with cancer cell membranes. Among these is the sinking raft model, in which peptides bind to the plasma membrane, resulting in the formation of transient pores [130]. The molecular electroporation model is another example; while not fully understood, this model involves the high charge density on the peptides providing an electrostatic potential that, upon interacting with the plasma membrane, results in electroporation and pore formation followed by cell death [130]. Cecropin A and B, as well as high concentrations of HNP1, HNP2 and HNP3, express their action through this latter model [29,30,59]. In the case of the last three peptides, the suppression of DNA synthesis in renal cell carcinoma lines causes reduced cell viabilities [131], while membrane permeabilization occurs through forming voltage-dependent, ion-permeable channels [132]. These three peptides may also induce DNA damage, as single-strand DNA breaks were detected in treated cells [133]. HNP-1 and HNP-3 may also disrupt neovascularization during tumor development and inhibit the proliferation of endothelial cells induced by vascular endothelial growth factor [134]. However, they were shown not to be tumor-selective, causing lysis of normal human leukocytes, epithelial cells and fibroblasts [135,136]. It has also been reported that their cytotoxicity can be serum-inhibited [137].

Another minor mechanism for ACPs membrane interaction is the aggregate channel model, where peptides form clusters on the plasma membrane by binding to the phospholipidic heads. The formed aggregates associate with water molecules, forming channels through which ions and larger molecules can pass without significant depolarization or destruction of the plasma membrane [138]. Magainins 2, A, B and G, at high concentrations, are reported to exhibit their anticancer activities through the formation of ion-conducting pores in cell membranes [62,67]. The anticancer activities of melittin are thought to involve the hyperactivation of phospholipase A2, an influx of Ca^+2^ and the subsequent destruction of the transformed cells, as in cells with ras overexpression [139,140]. Another reported mechanism involves the transient activation of endogenous phospholipase D, which leads to a signal transduction pathway, in turn promoting the membrane permeability of the peptide, as in U937 human monocytic leukemia cells [141].

The peptide-induced lipid segregation and the leaky-slit models play a limited role in ACPs interactions with membrane proteins. In the former, peptides binding to cell membranes lead to the grouping of anionic lipids into separate peptide–lipid domains and the segregation of zwitterionic lipids. The resulting rearrangement of the membrane layers affects cell viability and survival [116,142]. In the latter model, peptides bind to the membrane lipids forming linear amphipathic matrices, with the hydrophobic regions facing the double layer. The resulting highly positive curvature adopted by the lipids causes the formation of transient toxic fibrillar oligomers, the so-called leaky slits, that increase cell membrane permeability [142].

The classical and lysis-mediated complement pathways are other mechanisms adopted by by some ACPs such as Tachyplesin I to exhibit their anticancer activities. This mechanism involves binding to over-expressed hyaluronan in human TSU prostate carcinoma cells and to the C1q in human serum [143]. Tachyplesin I also adopts a non-cytolytic mechanism by inducing tumor cell differentiation, thereby reversing the malignant phenotype. This is manifested in the decreased expression of tumor-associated antigens (α-fetoprotein, proliferating cell nuclear antigen), modulation of differentiation-associated enzyme expression (γ-glutamyltransferase, tyrosine aminotransferase), decreased expression of the c-myc oncogene and increased expression of the tumor suppressor gene p21WAF1/CIP1, as observed in the peptide-treated SMMC-7721 human hepatoma cell and BGC-823 human gastric adenocarcinoma cell cultures [144,145].

The induction of syndecan-1 expression is another anticancer mechanism adopted by PR-39, as evident in hepatocellular carcinoma cell lines treated by this ACP [146]. PR-39 enters eukaryotic cells through a receptor-mediated process without permeabilizing the plasma membrane [46]. Within the cytosolic compartment, the NH_2_-terminal Arg residues in PR-39 form complexes with multiple SH3-containing cytoplasmic proteins, including the signaling adaptor protein p130Cas and the p85α regulatory subunit of phosphatidylinositol 3-kinase [46,147,148]

### 3.2. Non-Membrane Interactions

Apart from membrane interactions, some ACPs can act through angiogenesis inhibition, tumor apoptosis induction, essential cell protein targeting or immune cell recruitment [149]. The peptides P9, P12 and SP5031 were shown to cause angiogenesis inhibition due to interference with growth factor receptors [150,151,152]. ACPs, such as bovine lactoferricin, can penetrate into the intracellular compartment to reach the mitochondria, resulting in programmed cell death [86,90,153,154]. Some ACPs interfere with functional proteins to inhibit tumor genesis and progression, such as human LL-37, which inhibits proteasomes in gastric cancer cells [155,156]. Other ACPs, such as LTX-315 and LTX-401, can trigger immunogenic events against tumor cells [157,158]. LTX-315, for example, causes immunogenic cell death through the infiltration of T-lymphocytes and myeloid cells, which results in triggering a local inflammatory response. This can also release the inflammatory cytokine HMGB1 and ATP through transient focal necrosis, in addition to activating the apoptotic lysosome caspase-3 [36,159,160]. LTX-401, on the other hand, causes oncolytic necrosis that can promote the combined therapeutic effect of immunotherapies [158].

## 4. Effects of Hypoxia, pH and Enzyme Activation on ACPs

Hypoxia is a characteristic feature of the cancer tissue microenvironment. Fusing the trans activator of transcription with the hypoxia-inducible factor, HIF-1α protein, results in a species that is stable in a hypoxic environment but is degradable in healthy cell microenvironments; thus, selective penetration of cancer cell membranes is obtained [161].

Since cancer cells require high-energy uptake, they trigger a shift from oxidative to glycolytic metabolism. This is typically due to the upregulation of HIF-related genes, even when the physiological oxygen level is available, in a phenomenon known as the Warburg effect. This, in turn, results in increased cell accumulation of lactate acid [162,163,164]. Hypoxia also triggers increased activities of carbonic anhydrases, Na^+^/H^+^ exchangers, bicarbonate transporters and of indoleamine 2,3 dioxygenase, in addition to the accumulation of lactate acid all of these factors contribute to lowering the pH and are ultimately responsible for the acidic microenvironment that is always observed for cancer cells [165,166,167,168]. By taking advantage of this acidic microenvironment, pH low insertion peptides (pHLIPs) can be coupled to cell-penetrating peptides (CPPs) or to nano-carriers, where their acidic amino acid residues allow for their permeabilization of cancer cell membranes and their subsequent internalization [169,170]. The pH low insertion peptides are short unstructured peptides exhibiting weak interactions with cell membranes at neutral pH [171] but revert to helical conformation when the transmembrane domain becomes protonated at pH < 6.5, allowing the COOH-terminus to be inserted through the cell membrane [172].

Cancer cell membranes are externally coated with specific enzymes, not expressed on membranes of normal cells. These enzymes, such as metalloproteases, degrade protein structures in the extracellular matrix and therefore play a crucial role in tumor invasion and eventual metastasis. CPPs have their positively charged domain shielded with a negatively charged peptide domain through a peptide linker that can be cleaved by metalloproteases causing the activation of the CPP in the tumor microenvironment [173]. These cancer cell surface membrane receptors are therefore utilized for selective targeting by peptides or small ligands attached to CPPs such as brain and glioma targeting homing peptides and mitochondrial SS-peptides [174,175,176,177,178,179,180,181].

## 5. ACPs as Diagnostic Tools

### 5.1. Imaging Biosensors Employing ACPs

Radiolabeled antibodies have been recently widely applied with good outcomes. However, their high molecular weights which make them liable to sequestration by the reticuloendothelial and liver Kupffer cells, in addition to the long half-life of the isotopes employed, leading to their long elimination times from the body were shown to be disadvantages [182]. Radiolabeling of low molecular weight peptides that are typically less than 50 amino acid residues provides a better alternative as imaging tracers [183,184]. These peptides offer several advantages including their rapid uptake by the target tissues, good sensitivity in deep tissues, low bone marrow uptake, rapid plasma renal clearance and relatively low pharmacological dose to be administered, which make them suitable for imaging procedures. Analogs of regulatory peptides have also been applied for this purpose [185,186].

Examples of radiolabeled peptides and peptide analogs include somatostatin, a cyclic hormone expressed in the central and peripheral nervous systems, and cholecystokinin, a hormone present in the gastrointestinal tract and nervous system which is similar to gastrin both structurally and functionally. Further examples include the gastrin-releasing peptide and bombesin, which bind G-coupled receptors on prostate, breast, pancreatic and small-cell lung carcinoma cancer cells; agonistic peptide-receptor coupling binds to G-coupled proteins, internalizing the peptide-receptor complex, while antagonistic coupling acts externally on the cell membrane [187,188]; the secretin-like neuropeptides vasoactive intestinal peptide and pituitary adenylate cyclase-activating peptide; the glucagon-like peptide 1; and neurotensin [189].

Dipeptide nanoparticles have also been used as imaging and sensing probes. They are characterized by biocompatibility, visible fluorescence and photostability [190]. Fluorescent quantum dots and nanoclusters, such as gold nanoclusters, can also be conjugated with such peptide nano-assemblies for cancer imaging [191,192].

The linkage of dyes to the NH_2_-termini of pHLIPs allows for the labeling of tumor cells [193]. Changing one or two amino acid residues in the transmembrane domain of pHLIPs to alter their pH_50_, the pH at which 50% of the pHLIPs are inserted in the cancer cells, has been reported [194]. However, pHLIPs generally show relatively low tumor specificity and accumulate in the kidneys in pathologic and inflammation cases [195].

### 5.2. Non-Imaging Biosensing Techniques Employing ACPs

ACPs that are used in non-imaging biosensing techniques are typically short synthetic peptide ligands that are used for the detection of specific cancer markers [196]. In ELISA, an enzyme-linked conjugate and substrate are used to identify and quantify a specific target molecule in biological fluids through an antigen-antibody reaction with rapid low limit colorimetric detection with high specificity [197].

Synthetic peptides can also be used as probes in microarrays, where they are adsorbed on the surface of nitrocellulose-coated glass slides and are exposed to the specimen. A number of different unique peptide disease-specific biomarkers can be used in real time, randomly immobilized, to ensure equal accessibility to all antibodies on the peptide microarray during epitope mapping [198].

Binding of different peptides to biologically sensitive fluorophores creates various probes or molecular sensors [199]. In addition, peptides coupled to nanomaterials, such as Ag nanoparticles [200] and Au@Pt nanorods [201] have shown higher sensitivity, selectivity, stability as well as faster signal response [202,203,204,205]. This is of special significance since most diagnostic techniques target tumors but cannot efficiently detect circulating tumor cells due to their low concentration levels, heterogeneity in the blood sample, and non-specific binding of other normal cells, such as leukocytes [206].

## 6. Synthesis and Modification of ACPs

Solid phase peptide synthesis (SPPS) is the preferred synthesis method for small peptides containing less than 50 amino acid residues [207]. This can be achieved by means of fully automated peptide synthesizers employing non-proteinogenic amino acids with the possibility of applying post-transational modifications during the process. In this technique, the α-amino group and side chain are bound to temporary and semi-permanent protecting groups, respectively, while a polymer resin is coupled to the COOH-terminal residue from which the synthesis cycle is initiated proceeding towards the NH_2_–terminus, following the removal of the α-amino protecting group. The resin, typically stationed to the right for correct interpretation of sequence and stereochemistry, swells in the applied organic solvent to expand alongside the area of peptide growth, finally cleaving off the final product by means of a bifunctional linker that provides either a peptide acid or a peptide amide that is then isolated and characterized.

From a green chemistry perspective, SPPS has several disadvantages, including high solvent consumption and the use of hazardous chemicals [208]. On the other hand, SPPS meets many green criteria, such as a one-pot reaction, no mechanical losses, a simple work-up process, automatization and miniaturization, high yield and purity, and the potential for simultaneous synthesis of different peptides at the same conditions. Typically, two approaches can be applied in SPPS; these are the *tert*-butyloxycarbonyl (Boc)/benzyl and 9-fluorenylmethyloxycarbonyl (Fmoc)/tBu approaches. In the first of these strategies, the α-amino group is protected by the Boc group, which is later removed by trifluoroacetic acid in dichloromethane, while the side chain functional groups are protected by benzyl-base groups, which are later removed by hydrogen fluoride [209,210]. The second approach has the α-amino group being protected by the base labile Fmoc group, which is later removed typically by 20% piperidine in dimethylformamide, while the side chain functional groups are protected by the acid-labile *tert* -butyl or trityl-based groups, which are later removed by trifluoroacetic acid [211].

Liquid phase peptide synthesis, on the other hand, involves the synthesis of peptides in solution employing tags with different properties than the reagents and products to allow for its facile elimination. Several tag molecules have been used, such as polydisperse polyethylene glycol (PEG), monodisperse PEG, perfluoroalkyl substances, ionic liquids, polycarbons, hydrophobic polymers and phosphorus-containing tags [212].

Reconstructing the main chains of ACPs, or modifying their side chains, have been common approaches to enhancing their therapeutic effects [213]. The main chain transformation of ACPs through the replacement of the amino acids changes the activity and selectivity of the peptides by altering their net charges, hydrophilicities and conformations [214,215]. Changes involving non-natural amino acids allow for the synthesis of ACPs with a variety of physiochemical properties [216], including higher conformational flexibilities [217], higher metabolic stabilities [218] and more favored membrane interactions than ACPs with all-natural amino acids [219].

Incorporating cholesterol into the side chains of ACPs, on the other hand, facilitates their penetration into cancer cells by driving peptides self-assembly [220]. PEG coupling to side chain groups of ACPs increases their diameters, changing their physicochemical properties, extending their half-lives, improving their selectivity and reducing their toxicity towards normal cells [221,222]. Threonine, serine and tyrosine side chains, for example, can undergo phosphorylation [223,224], a post-synthesis modification, causing toxicity reduction towards normal cells [213]. Glycosidic bonds via glycosyltransferases may link sugars to specific amino acids on ACPs [225]; this sometimes results in the loss of their activity or function [226]. ACPs palmitoylation occurs through their reaction via a reversible thioester linkage with palmitate, a 16-carbon saturated fatty acid [227]. This reaction may improve either the ACPs selectivity or cytotoxicity to normal cells, but not both simultaneously [228].

## 7. Computational Approaches in ACPs Synthesis

**Figure 2:**
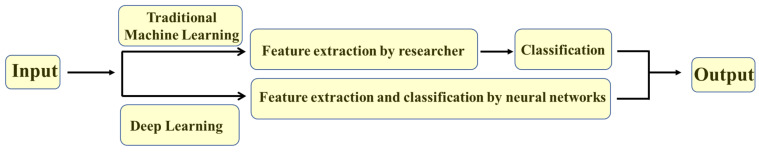
Schematic representation showing the main difference between traditional machine and deep learning approaches.

Traditionally, ACPs have been lab identified and synthesized through various experimental techniques; this process is cost, time and manpower intensive [20]. Recently, however, computationally supported studies to determine the potential interactions of identified ACPs with different proteins helped advance their development [229]. These techniques offer relatively accurate tools for predicting potential ACPs activities before starting in vitro evaluations, saving time, minimizing cost and maximizing output [20]. In the last decade, artificial intelligence (AI) has been used to develop efficient computational methods that can predict peptide sequences with high anti-cancer activities making the ACPs synthesis process faster and more targeted. Most of the AI-developed methods rely on using amino acid sequences of peptides [230,231,232]. Three AI approaches are currently being used in developing ACP predictors, traditional machine learning (ML), deep learning (DL)—which is a subset of ML—and hybrid methods using a combination of both, as shown in Figure 2 [230,231,232,233,234].

### 7.1. Traditional Machine Learning

In supervised machine learning, a labeled dataset of inputs and outputs is used to train the algorithm. This algorithm looks for a general formula that transforms the input into output. Patterns in data can be found using supervised machine learning algorithms for both continuous data and outputs with a categorical classification which is the regression. Unsupervised machine learning algorithms, on the other hand, use unlabeled data to find structure in the incoming data; this can be used to simplify or organize data [235]. AI computational methods of predicting ACPs rely on the use of a dataset consisting of the amino acid sequences of experimentally proven ACPs and non-ACPs. An initial step in the development of a statistical predictor is the selection or building of a valid benchmark dataset. A common dataset is utilized to examine the significance of the differences between the datasets, which would have two predefined classifications: anticancer and non-anticancer classes. These datasets are the base for training the machine learning algorithm and are a benchmark for testing various developed models [13,18,19].

In the feature extraction approach, datasets are subject to intensive data analysis that extracts different features linking them to the ACPs and non-ACPs datasets. The properties predicted by this approach include amino acid composition (AAC), dipeptide composition (DPC), atomic composition (AC), physicochemical properties (PCP), amino acid indices (AAINDEX and BLOSUM62) and amino acid Z-scales (which are five properties of amino acids as follows: Z1: lipophilicity, Z2: steric bulk and polarizability, Z3: polarity and charge, Z4 and Z5: electronegativity, heat of formation, electrophilicity and hardness) [13,236,237,238,239,240,241,242,243].

Some of the widely used ML algorithm classifiers are support vector machines (SVM), k-nearest neighbor (KNN), random forest (RF), ensemble classifiers with a clustering and dynamic selection (LibD3C), light gradient boosting machine (LightGBM), generalized neural network (GNN) and probabilistic neural network (PNN) [244,245,246,247]. The most widely used classifier, SVM, is a group of supervised learning techniques for classifying data, conducting regression analysis and identifying outliers. Because they select the decision boundary that optimizes the distance from the nearest data points of all the classes, SVM varies from other classification techniques. The maximum margin hyperplane is the name of the decision boundary produced by SVM [244]. In the RF algorithm, instead of relying on one decision tree, the random forest takes the prediction from each tree and bases its prediction of the final output on the majority votes of predictions [248].

**Figure 3:**
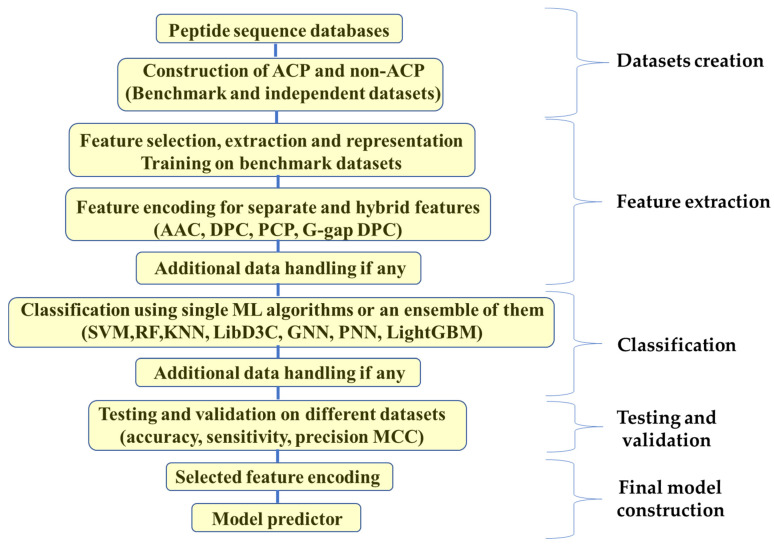
Schematic representation showing different steps of traditional machine learning approach for building a predictor for ACPs.

Classification is followed by a validation step to identify the accuracy, sensitivity, specificity and correlation coefficient of every studied case with a final suggested most accurate prediction model. Different cross-validation tests, including jackknife, k-independent and folding-based tests, have been employed in machine learning and pattern recognition to gauge the effectiveness of various predictors. Due to its exceptional outcomes, Jackknife stands out among all of these tests as being very effective and dependable, as presented in Figure 3 [249,250,251].

The SVM ML model was recently used with a balanced training dataset of 225 ACPs and 225 non-ACPs and applied on AAC and DPC as well as on a binary profile (BP) related order of amino acids features to develop AntiCP, an ML ACP model [242]. The second generation of this model, AntiCP 2.0, received a bigger training-balanced dataset of 861 ACPs and 861 non-ACPs and achieved accuracies of up to 88.8% [233]. A predictor model named MLACP [13] was developed using a Tyagi-B dataset as the training dataset and a screened HC dataset of Hajisharifi, Chen and LEE as the benchmarking dataset [243]. The RF and SMV ML classifiers with a 10-fold cross-validation method were recently employed, where the RF ML model showed an accuracy of 94.6% (0.885 Matthew’s correlation coefficient (MCC)) and 82.7% (0.674 MCC) on the HC and LEE dataset, respectively. In 2022, MLACP 2.0 was enhanced with large training and independent datasets, seven different ML classifiers on 17 different encoded features, with a final predictor model having nine features [252]. However, MLCP 2.0 has shown accuracy and MCC on an independent dataset of 76.5% and 0.513 respectively. The mACPpred machine learning model was proposed to predict ACPs with a balanced dataset of a total of 532 samples [253]. In this model, seven different encoding features are used to represent a peptide sequence and work with an SVM model to predict ACPs. The features extracted are AAC, DPC, composition–transition–distribution (CTD), quasi-sequence-order (QSO), amino acid index (AAIF), BP NC5 and conjoint triad (CTF). The best-developed predictor gave an accuracy of 91.7% and an MCC of 0.836. A highly advanced ML technique named DRACP, which reached an accuracy rate of 96%, is one in which the sequence and chemical properties of the amino acids were used to extract the feature of ACPs [254]. In this technique, the average 20 amino acid composition for a sequence was taken as a first feature. Then, based on the distribution of hydrophobic and hydrophilic residues, amino acids were divided into six groups according to their chemical properties, thus giving the second feature. These two features of ACPs were subsequently encoded using deep belief networks, and to identify the real ACPs, random relevance vector machines were employed. The effectiveness of this technique, which was shown to be reliable, was investigated on two different datasets. Many other ML predictor models were reported employing different datasets, features and ML methods, as summarized in Table 2. Some additionally developed ML models are the ACHP and the low-dimensional feature models [255,256].

### 7.2. Deep Learning (DL)

**Figure 4:**
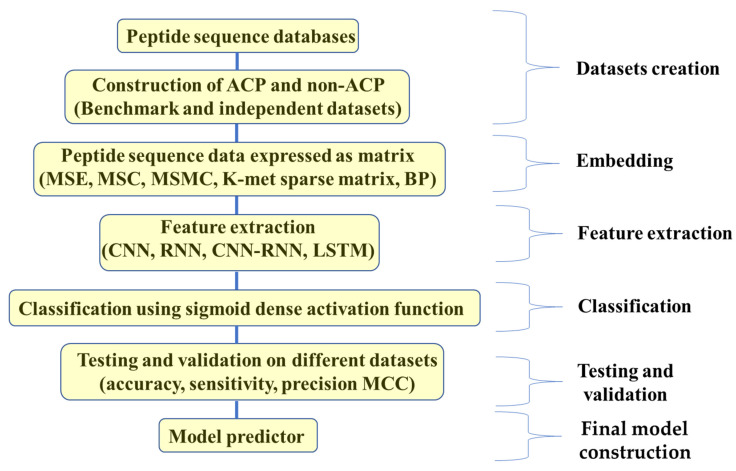
Schematic representation showing different steps of deep learning approach for building a predictor for ACP.

Several computational techniques have been proposed for the identification of ACPs, and an increasing number of machine learning algorithms, as previously discussed, are being used to build ACP predictors. Even though some of these methods have demonstrated comparatively good accuracy and robustness, selecting the proper features to capture the ACP sequences remains difficult for standard machine learning methods. DL algorithms are utilized to further increase the prediction accuracy and robustness to overcome this restriction. In DL, unlike traditional ML, the feature identification is carried out by means of AI rather than the researcher after a data embedding process in which the peptide sequence data are expressed as a matrix [19,233,234]. This is followed by the feature extraction step using DL algorithms such as convolutional neural network (CNN), long short-term memory (LSTM), attention model, recurrent neural network (RNN) and CNN-RNN [263,264]. CNN is a neural network architecture for DL that directly derives its learning from data, while RNN is a sequence model that performs input and output processing in units of sequence. Following DL feature extraction, classification is then carried out using the sigmoid dense activation function as shown in Figure 4 [234].

A long short-term neural network model (LSTM), that integrates binary profile information and a k-mer sparse matrix of the reduced amino-acid alphabet to successfully identify novel anticancer peptides was recently presented [234]. For the ACP-DL predictor, RNN was used to explicitly predict whether or not an input string of amino acids constitutes an ACP after each amino acid in each sequence is transformed into feature vectors before being fed into the LSTM. ACP-DL relies on a trained dataset ACP740 of 376 ACPs and 364 non-ACPs and an independent dataset ACP240 of 129 ACPs and 111 non-ACPs. A PTPD computational model utilizing a combination of Word2vec and CNN was shown to predict therapeutic peptides very effectively and is a notable recent advance [265]. In addition to extracting and combining various aspects, such as sequence, physicochemical and evolutionary-based features, in an interactive way for ACP identification, a new multi-headed deep convolutional neural network called ACP-MHCNN was introduced in 2020 [266]. In another approach, the DeepACP model, the predictors are built using three deep learning architectures; a CNN, a CNN-RNN and an RNN containing bidirectional long short-term memory cells (biLSTMs) [263]. The benchmark datasets of 250 ACPs and 250 non-ACPs tested against experimental findings demonstrate that the RNN architecture offers the best overall prediction performance. DeepACP results showed that biLSTMs in the RNN outperform other topologies. In order to choose the best architecture, a variety of alternate designs were created by changing the proportion of RNN cells, convolution kernels and network layers in CNNs. Benchmark and independent datasets used were similar to that of ACPred-FL. DeepACP performance was evaluated against some previously reported models on an independent dataset of 82 ACPs and 82 non-ACPs, as reported by Yu et al. [263].

The ACP-red LAF model developed in 2021 is a unique peptide representation-learning model based on learnable and adaptive embedding [264]. It uses a multi-sense and multi-scaled embedding technique that is an entirely end-to-end framework that does not require feature engineering and can automatically learn and extract sequential context features of ACPs. A multi-head attention method is applied in particular to help the model comprehend the discriminative features and enhance the feature representation capability in order to capture global information in ACP sequences. Compared to ML, ACP-red FL, a bigger and more refined balanced benchmark and independent datasets are used in DL ACP-red LAF. Upon testing ACP-red LAF on a balanced ACP-mixed-80 dataset, an accuracy of 81.15% and MCC of 0.633 was obtained compared to 74.32% and 0.519 as obtained for accuracy and MCC, respectively, by the AntiCP-DPC model. Several additional DL models are reported such as CL-ACP and more recently ACPNet [267,268].

### 7.3. Hybrid Approach and New Methods

Combining DL with traditional ML is performed through hybrid learning. A demonstration of data splitting of a total of 1722 samples balanced dataset, embedding and feature extraction were successfully handled by a DL hybrid learning process [19], whereas classification is handled by the traditional ML approach and testing performed on a 970 ACPs and 970 non-ACPs dataset showing an accuracy of 93.5%. On the other hand, the traditional ML approach performs data splitting and feature extraction with prediction performance improvement by means of data augmentation in ACP-DA [269]; while the multilayer perceptron (MLP) DL method performs classification. A newly introduced computational model for graph learning called ACP-GCN uses graph convolution networks to automatically and precisely predict ACPs [270]. In this approach, each peptide sample is represented as a graph, and for the first time, ACPs prediction is treated as a graph classification task. The unique model xDeep-AcPEP developed in 2021 aimed to predict the biological activity of ACPs against six tumor cells, including breast, colon, cervix, lung, skin and prostate, using a deep learning technique based on CNN [271]. This study demonstrated that learning models employing multi-tasking outperform traditional single-tasking models in terms of predictor performance. The uniqueness of this study comes from the fact that most of the prediction models of ACPs were made to sort peptides based on their amino acid sequences. On the other hand, the prediction was seen in this study as a multiclass classification problem that divides peptides into active, moderately active and inactive categories.

## 8. Future Prospects

Cancer treatment involves several modalities, such as surgery, chemotherapy, immunotherapy and radiotherapy. Despite such varied treatment options, cancer remains a leading cause of death globally. This highlights some inadequacies of such treatment options, which are accentuated when considering that surgery is not effective unless at an early stage and carries the risk of triggering metastasis, while chemotherapeutics generally lack specificity and may trigger cancer resistance. ACPs have shown promising performance both as diagnostic and therapeutic tools in terms of efficiency and specificity. ACPs, having originated from AMPs, carved out a distinct therapeutic role by extending our abilities in molecular targeting. The high cost of large-scale production of ACPs, their susceptibility to proteolytic cleavage and concerns about the usage of particular ACPs with sequences similar to those of human and natural AMPs due to the potential compromising of the human immune system and the possible subsequent threat to public health, are all significant drawbacks to ACPs large scale adoption. However, since ACPs are typically not targeted to specific extracellular or intracellular receptors, their use many inhibit many resistance mechanisms. This combined with their demonstrated cytotoxic efficacies against a variety of cancer types and will ensure their continued presence and development in the therapeutic anti-cancer arsenal. The success of ACPs depends on their sequences, secondary structures, net charges, amphipathicities, oligomerization abilities and high serum stabilities. Despite the absence of clear standards for ACPs design, an improved understanding of structures-activities relationships enhanced by innovative molecular representations and advanced computational approaches may provide valuable tools for advancing ACPs to theranostic success.

## Figures and Tables

**Table 1:** Summary of ACPs discussed in this review.

Peptide	Source	Primary Amino Acid Sequence ^a^	Class	Net Charge ^b^	Anticancer Mechanism	Reference
Aurein 1.2	*Litoria raniformis*	GLFDIIKKIAESF	α-Helix	+1	Barrel-stave pore mechanism	[25]
BMAP-27	*Bos taurus*	GRFKRFRKKFKKLFKKLSPVIPLLHL	α-Helix	+10	Membranolytic	[26]
BMAP-28	*Bos taurus*	GGLRSLGRKILRAWKKYGPIIVPIIRI	α-Helix	+7	Membranolytic	[27]
Brevinin	*Limnonectes fujianensis* frog	KLKNFAKGVAQSLLNKASCKLSGQC	Mixed α-Helix, β-sheet and random coil	+5	Lysosomal death pathway and autophagy-like cell death through depolarizing the transmembrane potential of cancer cells	[28]
Cecropin A	Silk moth *Hyalophora cecropia*	KWKLFKKIEKVGQNIRDGIIKAGPAVAVVGQATQIAK	α-Helix	+7	Membranolytic Apoptosis inducer	[29]
Cecropin B	Silk moth *Hyalophora cecropia*	KWKVFKKIEKMGRNIRNGIVKAGPAIAVLGEAKAL	α-Helix	+8	Tumor growth inhibition using pore formation and apoptosis	[30]
Citropin 1.1	*Litoria citropa* frog	GLFDVIKKVASVIGGL	α-Helix	+2	Carpet model of membrane disruption	[31,32]
D-K6L9	Synthetic	LKLLKKLLKKLLKLL	α-Helix	+3	Reduce neovascularization through cell membrane depolarization	[33]
Gaegurins	*Rana rugose* frog	Gaegurin 5: FLGALFKVASKVLPSVKCAITKKC	α-Helix	+4	Destruction of cell membranes through a carpet-like model and/or barrel-stave model	[34,35]
		Gaegurin 6: FLPLLAGLAANFLPTIICFISYKC				
HMGB1	*Homo sapiens*	GRRRRSVQWCAVSQPEATKCFQWQRNMRKVRGPPVSCIKRDSPIQCIQA	α-Helix	+9	Immature dendritic cells activation and tumor-specific cytotoxic generation	[36,37,38]
HNP-1, HNP-2 and HNP-3	*Homo sapiens*	HNP-1: ACYCRIPACIAGERRYGTCIYQGRLWAFCC	β-Sheet	+3	Membranolytic Antiangiogenic ^c^ Cytolytic activity	[39]
		HNP-2: CYCRIPACIAGERRYGTCIYQGRLWAFCC				
		HNP-3: DCYCRIPACIAGERRYGTCIYQGRLWAFCC				
hBD3	*Homo sapiens*	GIINTLQKYYCRVRGGRCAVLSCLPKEEQIGKCSTRGRKCCRRKK	Mixed	+11	Binding to the phosphatidylinositol 4,5-bisphosphate	[40]
LfcinB *	Mammalian lactoferrin	FKC1RRWQWRMKKLGAPSITC1VRRAF	β-Sheet	+8	Membranolytic Apoptosis inducer Antiangiogenic	[41]
LL-37 *	*Homo sapiens*	LLGDFFRKSKEKIGKEFKRIVQRIKDFLRNLVPRTES	α-Helix	+6	Toroidal pore formation	[42]
Magainin 2 *	*Xenopus laevis* frog	GIGKFLHSAKKFGKAFVGEIMNS	α-Helix	+3	Formation of pores on cell membranes Apoptosis	[43]
Melittin *	Venom of the European honeybee *Apis mellifera*	GIGAVLKVLTTGLPALISWIKRKRQQ	α-Helix	+6	Destabilizes the membrane through the barrel stave mechanism PLA2 ^d^ activator PLD ^e^ activator	[44]
P18	Synthetic hybrid	KWKLFKKIPKFLHLAKKF-NH_2_	α-Helix	+7	Membranolytic	[38,45]
PR-39	Porcine small intestine and neutrophils	RRRPRPPYLPRPRPPPFFPPRLPPRIPPGFPPRFPPRFP	Linear	+11	Induces syndecan-1 expression	[46,47]
Tachyplesin I *	*Tachypleus tridentatus* crab	KWC1FRVC2YRGIC2YRRC1R	β-Sheet	+6	Binds hyaluronan and activates complement (C1q) Antiangiogenic ^c^ Induces cancer cell differentiation	[48]

^a^ Amino acid sequences are given in one-letter codes. Subscripts indicate pairings of Cys residues that form disulfide bonds. Boldface indicates cationic amino acid residues. ^b^ At neutral pH. ^c^ Suggested activity ^d^ Phospholipase A2. ^e^ Phospholipase D. * Naturally occurring cationic antimicrobial peptides with anticancer activities.

**Table 2:** Some of the reported traditional ML predictor models showing their types of benchmark and independent datasets, features extracted, classifiers used, accuracy and MCC.

	Benchmark Dataset	Independent Dataset	Features	Classifier	Accuracy (%)	MCC	Reference
ACPP	SA_TRAIN	Balanced randomly generated peptides SA_IND	Protein-relatedness measures, including compositional, centroidal and distributional measures of amino acid residues	SVM	96	0.92	[257]
iACP	Hajisharifi et al. [243]	Balanced 300 peptides	One gap DPC	SVM	92.67	0.85	[258]
iACP-GAEnsC	Hajisharifi et al. [243]	NA	Pseudo g-Gap DPC	Ensemble method (SVM/RF/PNN/KNN/GRNN)	96.45	0.91	[259]
			Amphiphilic pseudo amino acid composition				
			Reduce amino acid alphabet composition				
ACPred	Hajisharifi et al. [243]	Balanced 205 peptides	AAC	SVM/RF	95.61	0.91	[260]
			DPC				
			PCP				
			Pseudo AAC				
			Amphiphilic pseudo AAC				
ACPred-FL	balanced dataset ACP500	balanced dataset ACP164	Composition–Transition–Distribution	SVM	91.4	0.835	[261]
			AAC				
			G-gap DPC				
			Adaptive skip DPC				
			BP Features				
			Overlapping Property Features				
			Twenty-One-Bit Features				
Target ACP	Hajisharifi et al. [243]	Balanced 205 peptides	Composite protein sequence representation	SVM/KNN/RF	98.78	0.97	[262]
			Split AAC				
			Pseudo position-specific scoring matrix				

## Data Availability

All data used in this review are contained within the references listed.

## Abbreviations

AAC: amino acid composition; AAIF: amino acid index; AC: atomic composition; ACP: anticancer bioactive peptide; aCPP: activable cell penetrating peptides; AI: artificial intelligence; AMP: antimicrobial peptide; AUNCs: gold nanoclusters; bFGF: basic fibroblast growth factor; Boc: tert –butyloxycarbonyl; BP: binary profile; Cas: carbonic anhydrases; CNN: convolutional neural network; CPP: cell-penetrating peptide; CTC: circulating tumor cells; CTD: composition-transition-distribution; CTF: conjoint triad; DCM: dichloromethane; DL: deep learning; DPC: dipeptide composition; ELISA: enzyme-linked immunoassay; Fmoc: fluorenylmethyloxycarbonyl; GLP-1: glucagon-like peptide 1; GNN: generalized neural network; GRP: gastrin-releasing peptide; hBD3: Human β-defensin 3; HF: hydrogen fluoride; HIF: hypoxia-inducible factor; HMGB1: High mobility group box protein 1; HNP: human neutrophil peptide; ICD: immunogenic cell death; IDO: Indoleamine 2,3 Dioxygenase; KNN: k-nearest neighbor; LfcinB: Lactoferricin B; LibD3C: ensemble classifiers with a clustering and dynamic selection; LightGBM: light gradient boosting machine; LL-37: Leucine leucine-37; LPPS: liquid phase peptide synthesis; LSTM: long short-term memory; MCC: Matthew’s correlation coefficient; ML: machine learning; MSC: multiscaled embedding; MSE: multisense embedding; MSMC: multisense-scaled embedding; NT: neurotensin; PACAP: pituitary adenylate cyclase-activating peptide; PCP: physicochemical properties; PEG: polyethylene glycol; pHLIPs: pH low insertion peptides; PLA2: Phospholipase A2; PLD: Phospholipase D; PNN: probabilistic neural network; QSO: quasi-sequence-order; Ras: rat sarcoma virus; RF: random forest; RNN: recurrent neural network; ROS: reactive oxygen species; SPPS: Solid phase peptide synthesis; SVM: support vector machines; Tat: trans activator of transcription; TFA: trifluoroacetic acid; VEGF: vascular endothelial growth factor; VIP: vasoactive intestinal peptide.
